# Postoperative ulnar neuropathy: a systematic review of evidence with narrative synthesis

**DOI:** 10.1016/j.bja.2023.04.010

**Published:** 2023-05-15

**Authors:** David W. Hewson, Thomas Kurien, Jonathan G. Hardman

**Affiliations:** 1 Department of Anaesthesia and Critical Care, Queen's Medical Centre, Nottingham University Hospitals NHS Trust, Nottingham, UK; 2 Academic Unit of Injury, Recovery and Inflammation Sciences, School of Medicine, University of Nottingham, Nottingham, UK; 3 Department of Trauma and Orthopaedic Surgery, Queen's Medical Centre, Nottingham University Hospitals NHS Trust, Nottingham, UK

**Keywords:** anaesthesia, patient positioning, patient safety, peripheral nerve injury, ulnar nerve, ulnar nerve compression syndromes

## Abstract

**Background:**

Postoperative ulnar neuropathy (PUN) is an injury manifesting in the sensory or motor distribution of the ulnar nerve after anaesthesia or surgery. The condition frequently features in cases of alleged clinical negligence by anaesthetists. We performed a systematic review and applied narrative synthesis with the aim of summarising current understanding of the condition and deriving implications for practice and research.

**Methods:**

Electronic databases were searched up to October 2022 for primary research, secondary research, or opinion pieces defining PUN and describing its incidence, predisposing factors, mechanism of injury, clinical presentation, diagnosis, management, and prevention.

**Results:**

We included 83 articles in the thematic analysis. PUN occurs after approximately 1 in 14 733 anaesthetics. Men aged 50–75 yr with pre-existing ulnar neuropathy are at highest risk. Preventative measures, based on consensus and expert opinion, are summarised, and an algorithm of suspected PUN management is proposed, based upon the identified literature.

**Conclusions:**

Postoperative ulnar neuropathy is rare and the incidence is probably decreasing over time with general improvements in perioperative care. Recommendations to reduce the risk of postoperative ulnar neuropathy are based on low-quality evidence but include anatomically neutral arm positioning and padding intraoperatively. In selected high-risk patients, further documentation of repositioning, intermittent checks, and neurological examination in the recovery room can be helpful.

**Editor's key points**

• Preventing ulnar and other pressure-related nerve injuries is an important part of safe anaesthetic care for an unconscious patient.
• This evidence synthesis suggests perioperative ulnar nerve injury is rare, but good anaesthetic care is likely to be an important reason for this.
• The authors provide useful guidance on the prevention and treatment of this important problem.

The prevention of physical injury to patients whilst under the effects of sedation, regional or general anaesthesia, is a fundamental duty of anaesthetists. Mechanical, thermal, and electrical injuries to soft tissues, including skin, muscles, and nerves, have all been described whilst patients undergo surgery. Injury to the ulnar nerve is a feared adverse event after anaesthesia and was traditionally viewed by clinicians, patients, and legal professionals as a preventable complication arising from surgical or anaesthetic care.^1^ The ascription of liability for ulnar nerve injury has often followed the legal doctrine of *res ipsa loquitur* (‘the thing speaks for itself’; in other words, that the event would ordinarily not occur in the absence of negligence), even though it has been acknowledged for at least 30 yr that postoperative ulnar neuropathy (PUN) is not always preventable.^2^ Although few cases of PUN progress to successful civil litigation, during their career anaesthetists are very likely to be asked to review cases of possible PUN and must therefore understand how to assess and manage the condition.

To understand and summarise the current literature on PUN, we have conducted a systematic literature review with a narrative synthesis. Our objectives were to define the condition and describe its incidence, predisposing factors, mechanism of injury, clinical presentation, diagnosis, management, and prevention.

## Methods

In this review, we sought to identify articles describing PUN. After a systematic literature search, we used narrative synthesis to summarise current understanding of the condition and derive implications for practice and research on this topic. The review methodology was informed by the *Guidance on the Conduct of Narrative Synthesis in Systematic Reviews*,^3^ including the use of thematic analysis to identify the main themes and concepts across studies.

### Definitions

In the absence of consensus-derived definitions for terms used in this review, we have defined *perioperative* as meaning occurring at or around the time of surgery performed in the presence of an anaesthetist delivering monitored anaesthetic care, sedation, regional anaesthesia, general anaesthesia, or any combination of these. *Ulnar neuropathy* was defined as a new clinical or neurological abnormality in the expected motor or sensory distribution of the ulnar nerve along its course from origin at the medial cord of the brachial plexus to its most distal branches in the hand. The terms *ulnar nerve palsy* and *ulnar nerve injury* were considered within the search strategy and results analysis as synonymous, acknowledging the variation in terms used in the literature to describe the phenomenon in question. The Seddon^4^ and Sunderland^5^ classifications of peripheral nerve injuries have been extensively described before and will not be reproduced in the current work. For inclusion in this review, we considered axonotmesis, neurotmesis, and neuropraxia (temporary segmental demyelination) to constitute PUN.

### Eligibility criteria

#### Inclusion criteria for studies were:

(i) Primary (case reports, laboratory studies, observational or non-experimental studies, and prospective trials) or secondary (systematic reviews with or without meta-analyses) research or opinion pieces (editorials or journal correspondence) addressing any or all of the following: incidence, predisposing factors, mechanism of injury, clinical presentation, diagnosis, management, or prevention of PUN
(ii) Reporting PUN in humans
(iii) Available in English language or with English language translation

### Search strategy

We performed a search of titles, abstracts, keywords, and medical subject headings terms from the following databases from inception to October 3, 2022: MEDLINE, EMBASE, Scopus, Web of Science, and Google Scholar. A grey literature search was conducted on the ProQuest Dissertations & Theses platform. To minimise bias in the return of Google Scholar search results, the search was conducted using a web browser in private browsing mode. The first 200 Google Scholar records were reviewed for inclusion.

The following search terms were applied with spelling wildcards, truncation, and Boolean operators: *ulnar*, *nerve*, *neuropathy*, *palsy*, *paralysis*, *dysfunction*, *injury*, *perioperative*, *intraoperative*, *postoperative*, and *anaesthesia*. The search was adapted for application to individual databases. The search strategy is provided as a supplementary file.

Literature reporting inadvertent direct surgical injury nerves was included in the review, as the investigation and management of such cases provide transferrable insights into the assessment and treatment of PUN.

### Data extraction and quality assessment

After automated exclusion of duplicate entries, titles and abstracts of returned studies were screened for full-text review for eligibility and subsequent inclusion. The reference lists of studies selected for full-text review were screened for additional articles not identified by the aforementioned search terms. Uncertainty regarding the inclusion of an article was discussed between two authors (DWH and JGH) and resolved by consensus between all authors. Data (article title, authors, year of publication, country of origin, study design, and initial thematic content analysis)^6^ were extracted and tabulated for all eligible papers.

Given the purposively broad inclusion criteria and variety of research formats returned by the search terms, the methodological index for non-randomised studies (MINORS) instrument was used to perform a quantitative quality assessment of included non-randomised observational studies.^7^ MINORS provides assessment of 12 methodological domains, each domain scoring 0 (not reported), 1 (reported but inadequate), or 2 (reported and adequate). These sum to a global ideal MINORS score of 16 for non-comparative studies and 24 for comparative studies. Comments relating to methodological weaknesses of specific studies or articles have been included in the narrative analysis.

## Results

**Fig 1:**
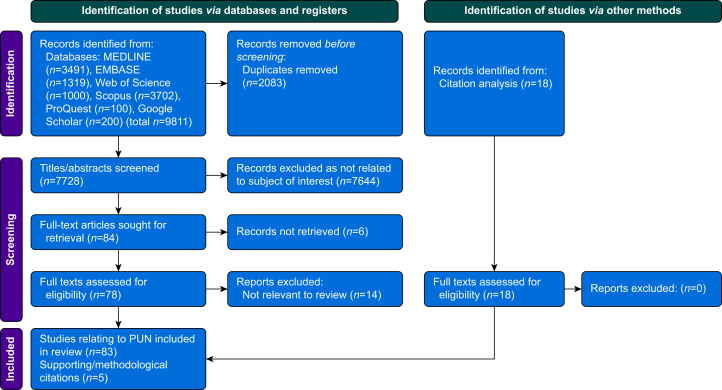
Preferred Reporting Items for Systematic Reviews and Meta-Analyses flow diagram^8^ of search results. PUN, postoperative ulnar neuropathy.

A Preferred Reporting Items for Systematic Reviews and Meta-Analyses flow diagram^8^ of search results is shown in Figure 1. The identified articles included in the final analysis, together with the global MINORS assessment of methodological quality for relevant studies, are listed in Table 1.

**Table 1:** Identified articles relating to PUN included in final thematic analysis with global MINORS assessment of methodological quality. MINORS, methodological index for non-randomised studies; N/A, not available; PUN, postoperative ulnar neuropathy.

Author(s); country of origin; year of publication	Study design	Thematic analysis subheading(s)	Number of patients identified with PUN/total sample size	Global MINORS score/total possible MINORS score
Kroll and colleagues^9^; USA; 1990	Retrospective or registry analysis	Incidence; prevention	N/A (closed claims analysis)	N/A
Warner and colleagues^10^; USA; 1999	Observational clinical study	Incidence; predisposing factors; clinical presentation	7/1502	13/16
Warner and colleagues^11^; USA; 1994	Retrospective or registry analysis	Incidence; predisposing factors; natural history	414/1 129 692	13/16
Pulos and colleagues^12^; USA; 2021	Retrospective or registry analysis	Incidence; predisposing factors	22/324 124	14/16
Seyfer and colleagues^13^; USA; 1985	Observational clinical study	Incidence	20/53	12/16
Merchant and colleagues^14^; Canada; 1990	Observational clinical study	Incidence	1/20	6/16
Chui and colleagues^15^; Canada; 2018	Retrospective or registry analysis	Incidence; mechanism of injury; prevention; diagnosis	N/A (closed claims analysis)	N/A
Alvine and Schurrer^16^; USA; 1987	Observational clinical study	Predisposing patient factors; diagnosis	17/6538	13/24
Cheney and colleagues^17^; USA; 1999	Retrospective or registry analysis	Predisposing patient factors	N/A (closed claims analysis)	N/A
Perreault and colleagues^18^; Canada; 1992	Review or opinion article	Predisposing patient factors; mechanism of injury; diagnosis; management	N/A	N/A
Contreras and colleagues^19^; USA; 1998	Laboratory/volunteer study	Predisposing patient factors	N/A	N/A
Shimokata and colleagues^20^; USA; 1989	Laboratory/volunteer study	Predisposing patient factors	N/A	N/A
Hattori and colleagues^21^; Japan; 1991	Laboratory/volunteer study	Predisposing patient factors	N/A	N/A
O'Driscoll and colleagues^22^; USA; 1991	Laboratory/volunteer study	Predisposing patient factors	N/A	N/A
Campbell and colleagues^23^; USA; 1991	Laboratory/volunteer study	Predisposing patient factors	N/A	N/A
Morell and colleagues^24^; USA; 2003	Laboratory/volunteer study	Predisposing patient factors	N/A	N/A
Jones^25^; UK; 1967	Case series or report	Predisposing patient factors; natural history	N/A	N/A
Casscells and colleagues^26^; USA; 1993	Observational clinical study	Predisposing patient factors; diagnosis	11/42	12/24
Schmitt and Muenster^27^; Germany; 2009	Review or opinion article	Predisposing patient factors	N/A	N/A
Welch and colleagues^28^; USA; 2009	Retrospective or registry analysis	Predisposing factors; prevention	N/A	N/A
Harding and Morris^29^; UK; 2003	Observational clinical study	Mechanism of injury	N/A	10/16
Khoo and colleagues^30^; USA; 1996	Review or opinion article	Mechanism of injury	N/A	N/A
Leffert and Dorfman^31^; USA; 1972	Case series or report	Mechanism of injury	N/A	N/A
Kurvers and Verhaar^32^; Netherlands; 1995	Retrospective or registry analysis	Mechanism of injury	N/A	N/A
Mulder and colleagues^33^; USA; 1961	Retrospective or registry analysis	Mechanism of injury	N/A	N/A
Platt^34^; UK; 1926	Case series or report	Mechanism of injury	N/A	N/A
Sharp and colleagues^35^; USA; 2021	Review or opinion article	Mechanism of injury	N/A	N/A
Slobogean and colleagues^36^; Canada; 2010	Review or opinion article	Mechanism of injury	N/A	N/A
Rasulić and colleagues^37^; Serbia; 2017	Case series or report	Mechanism of injury	N/A	N/A
Thorkildsen and colleagues^38^; Norway; 2021	Case series or report	Mechanism of injury	N/A	N/A
Johnson and colleagues^39^; USA; 2015	Review or opinion article	Mechanism of injury	N/A	N/A
Swenson and colleagues^40^; USA; 1998	Laboratory/volunteer study	Mechanism of injury	N/A	N/A
Hutchinson and McClinton^41^; USA; 1993	Laboratory/volunteer study	Mechanism of injury	N/A	N/A
Cameron and Stewart^42^; Canada; 1975	Case series or report	Mechanism of injury; presentation; natural history; diagnosis	N/A	N/A
Moore and colleagues^43^; New Zealand; 2014	Laboratory/volunteer study	Mechanism of injury	N/A	N/A
Murphy and Devers^44^; USA; 1974	Review or opinion article	Mechanism of injury	N/A	N/A
Ashenhurst^45^; Canada; 1962	Laboratory/volunteer study	Mechanism of injury	N/A	N/A
Payan^46^; Denmark; 1970	Case series or report	Mechanism of injury	N/A	N/A
Childress^47^; USA; 1956	Laboratory/volunteer study	Mechanism of injury	N/A	N/A
Billmann and colleagues^48^; Germany; 2014	Observational clinical study	Mechanism of injury	N/A	10/16
Vanderpool and colleagues^49^; USA; 1968	Case series or report	Mechanism of injury	N/A	N/A
Feindel and Stratford^50^; Canada; 1958	Case series or report	Mechanism of injury	N/A	N/A
Bagatur and colleagues^51^; Turkey; 2016	Case series or report	Mechanism of injury	N/A	N/A
Granger and colleagues^52^; Grenada; 2017	Review or opinion article	Mechanism of injury	N/A	N/A
Adelaar and colleagues^53^; USA; 1984	Case series or report	Mechanism of injury	N/A	N/A
Sunderland^54^; Australia; 1945	Review or opinion article	Mechanism of injury	N/A	N/A
Zylicz and colleagues^55^; Netherlands; 1984	Case series or report	Mechanism of injury	N/A	N/A
Gertel and Shapira^56^; Israel; 1987	Case series or report	Mechanism of injury	N/A	N/A
Terhoeve and colleagues^57^; USA; 2022	Case series or report	Mechanism of injury	N/A	N/A
Anonymous^58^; USA; 2018	Clinical practice guideline	Prevention	N/A	N/A
Sy^59^; USA; 1981	Case series or report	Prevention	N/A	N/A
Liang^60^; USA; 1997	Case series or report	Prevention	N/A	N/A
Warner and colleagues^61^; USA; 2000	Observational clinical study	Prevention; incidence	2/986	13/16
Grant and colleagues^62^; USA; 2019	Retrospective or registry analysis	Prevention; natural history; management	N/A (closed claims analysis)	N/A
Beekman and colleagues^63^; Netherlands; 2009	Observational clinical study	Clinical presentation; diagnosis	N/A	13/16
Copp^64^; UK; 1965	Case series or report	Clinical presentation	N/A	N/A
Barr^65^; USA; 1974	Review or opinion article	Natural history	N/A	N/A
Caplan and colleagues^66^; USA; 1994	Review or opinion article	Diagnosis	N/A	N/A
Hewson and colleagues^67^; UK; 2018	Review or opinion article	Diagnosis	N/A	N/A
Upton and McComas^68^; Canada; 1973	Case series or report	Diagnosis	N/A	N/A
Molinari and Elfar^69^; USA; 2013	Review or opinion article	Diagnosis	N/A	N/A
Bage and Power^70^; UK; 2021	Review or opinion article	Diagnosis; management	N/A	N/A
Read^71^; UK; 1988	Review or opinion article	Diagnosis	N/A	N/A
Pulos and colleagues^72^; USA; 2019	Review or opinion article	Diagnosis; management	N/A	N/A
Cesmebasi and colleagues^73^; USA; 2015	Case series or report	Diagnosis	N/A	N/A
Staff and colleagues^74^; USA; 2010	Case series or report	Diagnosis	N/A	N/A
British Orthopaedic Association^75^; UK; 2012	Clinical practice guideline	Management	N/A	N/A
Regional Anaesthesia UK^76^; UK; 2021	Clinical practice guideline	Management	N/A	N/A
Ross^77^; UK; 2017	Review or opinion article	Management	N/A	N/A
Wojtkiewicz and colleagues^78^; USA; 2015	Review or opinion article	Management	N/A	N/A
Chui and colleagues^79^; Canada; 2021	Observational clinical study	Diagnosis	N/A	16/16
Hickey and colleagues^80^; USA; 1993	Case series or report	Diagnosis	N/A	N/A
Chui and colleagues^81^; Canada; 2017	Observational clinical study	Diagnosis	N/A	12/16
Chui and colleagues^82^; Canada; 2019	Observational clinical study	Diagnosis	N/A	12/16
Laughlin and colleagues^83^; USA; 2014	Case series or report	Diagnosis	N/A	N/A

### Incidence

The first description of postoperative peripheral nerve injury arising from anaesthesia was published in 1894,^84^ and ulnar neuropathy after anaesthesia was described in 1901.^85^ The ulnar nerve is disproportionately affected by perioperative neuropathy in comparison with other major peripheral nerves of the upper limb.^9^ In a prospective cohort study of 1502 adult patients undergoing noncardiac surgery under general, spinal, or regional anaesthesia and followed up with questionnaires and neurological examination for the first seven postoperative days, sensory or combined sensory/motor PUN was identified in seven patients: an incidence of 0.52% (95% confidence interval [CI]: 0.2–1.0%) or approximately one in 200 cases.^10^ A retrospective analysis of 1 129 692 episodes of noncardiac anaesthesia over the period 1957–91 identified an incidence of PUN with sustained motor impairment persisting more than 3 months of 0.037% (95% CI: 0.033–0.040%) or approximately one in 2500 cases.^11^ The disparity in the incidences reported in these studies may arise from their differing methodologies (prospective seeking of diagnoses *vs* retrospective interrogation of diagnostic coding) and the natural history of the condition (of neurological improvement over time), especially when the deficit was sensory only.

An updated retrospective analysis conducted by the same group on data from 324 124 patients undergoing noncardiac surgery between 2011 and 2015 demonstrated an incidence of sustained motor PUN of at least 2 months duration of 0.007% (95% CI: 0.004–0.010%) or approximately one in 14 733 cases.^12^ The authors propose that the decreasing incidence over time is attributable to earlier patient ambulation after surgery, shorter hospital stays, and implementation of best practice guidance on positioning for surgery (see section below, 'Prevention'). The seemingly low ‘headline’ incidence derived from this sample of unselected patients undergoing noncardiac surgery hides the fact that certain patient characteristics place them at significantly higher risk of PUN (see section below, ‘Predisposing patient factors’).

Cardiac surgery may carry a higher risk of PUN compared with noncardiac surgery, with an incidence of up to 37.7% demonstrated in a prospective work seeking evidence of injury in patients after median sternotomy and cardiopulmonary bypass (CPB).^13^ When tested preoperatively, approximately one-third of patients undergoing cardiac surgery have subclinical ulnar neuropathy,^14^ which may predispose patients to manifest clinically relevant PUN postoperatively. Unlike noncardiac surgery, it is likely that abnormalities of ulnar nerve conduction after cardiac surgery at least partially reflect injury at the level of the brachial plexus attributable to surgical tissue retraction, rather than the more peripheral nerve injuries associated with noncardiac surgery. The contributory role of periods of global hypoperfusion, hypotension, or hypothermia, associated with cardiac surgery and CPB to a greater extent than noncardiac surgery, is also unclear.

A recent analysis of peripheral nerve injuries recorded on the ASA Closed Claims Database demonstrated PUN to be the second most frequent injury (accounting for 30% of peripheral nerve injuries), with brachial plexus-level injuries accounting for 36% of cases. Median and radial nerve injuries were much less frequently recorded (10% and 8%, respectively).^15^

### Predisposing patient factors

Table 2 lists patient and perioperative risk factors for the development of PUN. Men aged 50–75 yr appear to be at highest risk and may be at approximately three times higher risk than females.^10^^,^^16^ Three-quarters of ASA Closed Claims cases of PUN are reported in men.^17^ This gender difference has been explained by the propensity of the male cubital tunnel to serve as a site of ulnar injury^18^ because of a larger coronoid process, thicker retinaculum, and relative paucity of overlying adiposity.^19^, ^20^, ^21^, ^22^, ^23^ Experimental application of direct pressure over the ulnar nerve shows a sex-specific difference in unmyelinated C-fibre susceptibility to pressure injury, with males demonstrating a greater effect of compression than females (response ratio 1.7 [95% CI: 1.2–2.4]).^24^ Right and left nerves are believed to be affected equally. In one retrospective review of over one million patients undergoing noncardiac surgery, prolonged hospitalisation, greater than 14 days, was associated with PUN,^11^ suggesting that factors beyond the episode of direct anaesthetic care may play a role in the aetiology of the condition.

**Table 2:** Summary of reported risk factors for development of postoperative ulnar neuropathy.

Category	Risk factor
Patient	Male sex
	50–75 yr of age
	Chronic hypertension
	Diabetes mellitus
	Peripheral vascular disease
	Pre-existing peripheral neuropathy
	High body mass index
	History of cancer
	Tobacco use
	Chronic alcohol excess
Perioperative	Longer surgical duration
	Arms ‘tucked’ to torso
	Elbow flexion >90°
	Forearm pronation when patient supine
	Hospital length of stay >14 days

Pre-existing peripheral neuropathy is recognised as a risk factor for dysfunction in the ulnar nerve conduction after surgery.^25^^,^^26^ If a peripheral neuropathy is known to exist preoperatively, a neurological examination should be performed to document its extent and characteristics.^27^^,^^58^ Hereditary motor and sensory polyneuropathies, such as Charcot–Marie–Tooth and Dejerine–Sottas diseases, are possibly associated with a greater incidence of postoperative peripheral nerve injury.^18^ Larger BMI (odds ratio [OR] 1.67 per 5 kg m^−2^ increase; 95% CI: 1.16–2.42; *P*=0.006), longer surgical duration (OR 1.53; 95% CI: 1.18–1.99; *P*=0.001), history of cancer (OR 6.46; 95% CI: 1.64–25.49; *P*=0.008), and when arms are adducted in relation to the torso (‘tucked’) during surgery (OR 6.16; 95% CI: 1.85–20.59; *P*=0.003)^12^ increase the risk of PUN. Other predisposing factors proposed in the literature to be associated with an increased risk of perioperative nerve injury are hypertension, diabetes mellitus, tobacco use,^28^ peripheral vascular disease, and alcohol misuse.^58^

Because many of the aforementioned factors are elicited as part of preoperative anaesthetic assessment, anaesthetists are in an excellent position to undertake a patient-specific consent discussion, disclosing the likely risk of PUN and the steps taken perioperatively to mitigate risk (see section below ‘Prevention’).

### Mechanism of injury

Mechanistic insight into the pathophysiology of ulnar neuropathy can be derived from patients who do and do not undergo surgery. Compression neuropathy of the ulnar nerve at the elbow was first systematically described by Sir William Gowers^86^ in his 1886 book *A Manual of Diseases of the Nervous System*. Causes of non-surgical entrapment at the elbow include anatomical abnormalities, rheumatoid and osteoarthritis, medial epicondylitis, diabetes mellitus, trauma, and habitual leaning on the elbow arising from occupations.^29^, ^30^, ^31^, ^32^, ^33^, ^34^ In a number of cases, no single cause is found to explain the neuropathy, and such cases are usually described as idiopathic. As a specific phenomenon, postoperative ulnar nerve injury has been ascribed to direct nerve trauma before or during upper-limb surgery (e.g. supracondylar humeral fracture^35^, ^36^, ^37^ or implantable contraceptive device removal)^38^; trauma from peripheral nerve block; nerve infarction during transposition surgery; and, finally, arising from injudicious patient positioning under anaesthetic. Complete section of the ulnar nerve is unlikely to arise solely from anaesthetic care.^18^ In one analysis of iatrogenic peripheral nerve injuries presenting to a single neurosurgical service over a 10 yr period, direct intraoperative damage incurred by inadvertent surgical trauma to the ulnar nerve during surgery for supracondylar humeral fracture was the most frequent mechanism of iatrogenic intraoperative ulnar injury.^37^ Unlike PUN, in 85% of such cases, total ulnar nerve discontinuity was demonstrated.

Widely accepted mechanistic explanations for ulnar neuropathy are stretch or compression of the nerve,^15^ causing physical distortion to normal nerve architecture and disruption to the vasa nervorum.^39^ Resultant nerve ischaemia disrupts normal axonal transmission and causes neuropathy. There is evidence that the ulnar nerve is more sensitive to global ischaemia than other nerves. In one study of somatosensory evoked potentials (SSEPs) in 10 healthy volunteers undergoing general anaesthesia, the change in ulnar nerve signal amplitude brought about by brachial artery occlusion was significantly greater compared with both median and radial nerves.^40^ Similarly, in awake volunteers subjected to upper-limb tourniquet ischaemia, paraesthesia in an ulnar distribution is observed before median or radial nerve symptoms (although the degree to which this effect is observed as a result of solely ischaemia, rather than combined ischaemia and direct compression, is uncertain).^41^ The topography of the ulnar nerve at the elbow (with relatively little epineural tissue and a superficial position) may increase susceptibility to injury.^42^ In healthy volunteers, reduced blood flow is observed in the posterior ulnar recurrent artery (supplying the ulnar nerve at the cubital tunnel) when the elbow is flexed to 120°. This could contribute to nerve ischaemia and subsequent injury during excessive or prolonged elbow flexion during anaesthesia.^43^

Stretch of the ulnar nerve is brought about by prolonged flexion of the elbow beyond 90°—a commonly observed phenomenon in awake or drowsy individuals, where relief from paraesthesia is brought about by elbow extension. Inappropriate stretch may occur in both prone and supine patient positions, as dictated by the needs of surgery.^44^ Hypermobility of the ulnar nerve manifests as partial or complete dislocation of the nerve outside the cubital groove; it is present in approximately 20% of patients^45^, ^46^, ^47^ and may increase risk of injury by worsening compressive forces when the nerve dislocates.^18^^,^^48^

Compression of the ulnar nerve commonly occurs at the cubital tunnel.^49^^,^^50^ The cubital tunnel transmits the ulnar nerve into the forearm; it is a fibro-osseous space at the posteromedial aspect of the elbow. The tunnel is bordered medially by the medial epicondyle of the humerus and laterally by the olecranon of the ulna. The floor is formed by the elbow joint capsule and the medial collateral ligament of the elbow. The roof is formed by the cubital tunnel retinaculum (the arcuate ligament of Osborne)—a 4-mm-wide band of fascia running from medial epicondyle to olecranon between the two heads of flexor carpi ulnaris.^22^ The retinaculum functions to hold the ulnar nerve in position behind the medial epicondyle. This retinaculum is sometimes supplemented by the atavistic anconeus epitrochlearis muscle, an anatomical variant present in approximately a third of individuals.^51^ During elbow flexion, the tunnel changes from oval to elliptical shape in cross section. At full elbow extension, the retinaculum is lax, whereas in elbow flexion to 90° the retinaculum becomes taut and reduces the capacity of the tunnel.^52^^,^^53^ A study performed in 27 cadaveric specimens revealed four anatomical retinacula subtypes: Type 0, absent retinaculum (4%); Type 1a, present retinaculum taut in full flexion (63%); Type 1b, present retinaculum taut in less-than-full flexion (22%); and Type 2, absent retinaculum replaced by the anconeus epitrochlearis muscle (11%).^22^ The authors postulated that these anatomical variations may explain why certain individuals develop ulnar neuropathy, whereas others do not. Type 0 retinaculum (i.e. absent) allows medial ulnar nerve displacement to the tip of the medial epicondyle and exposing it to external compression; Type 1b may predispose to dynamic compression with only moderate degrees of elbow flexion, and Type 2 may lead to compression attributable to the bulk of the anconeus muscle. Although the authors were able to correlate retinaculum variants with cadaveric evidence of chronic ulnar compression, whether these variants are neatly associated with *in vivo* clinical presentations of ulnar neuropathy remains unknown.^51^ Within the cubital tunnel, the sensory fibres of the ulnar nerve are typically more superficial than motor components, and it is theorised that sensory components are therefore more vulnerable to external compression.^54^

Certain perioperative conditions increase the susceptibility of the ulnar nerve to ischaemic damage, including hypothermia and hypotension. Analysis of individual cases of PUN usually cannot identify with certainty a specific causative link between a single pre- or intraoperative clinical circumstance and subsequent presentation with neuropathy. By way of illustration, a published case series of eight patients developing ulnar neuropathy after renal transplantation at a single centre over a short period of time speculatively identified multiple possible causative factors, including external pressure on the cubital tunnel applied whilst operating by the surgeon, blood pressure cuff monitoring pressure, venous congestion induced by arteriovenous shunting, and pre-existing but subclinical uraemic polyneuropathy.^55^ Occasionally, a definite (and usually avoidable) single causative factor is identified. For example, the electrode from a cutaneous nerve stimulator (to assess neuromuscular block), applied to the ulnar nerve at the elbow and the arm inappropriately positioned in such a way that compressive force was transmitted from the device to the cubital tunnel, leading to a PUN after appendicectomy.^56^ A 1990 report generated from the ASA Closed Claims Database identified a specific injurious mechanism for PUN in only 6% of cases.^9^ Uncertain aetiology continues to typify this condition: a recent report of isolated ulnar neuropathies presenting after COVID-19 infection acknowledged this uncertainty and speculated that patient positioning during acute illness, the prothrombotic underlying disease process, direct virological effects, or immune-mediated nerve injury may have played a role in the genesis of the nerve injuries.^57^

### Prevention

In 2018, the ASA updated their *Practice Advisory for the Prevention of Perioperative Peripheral Neuropathies*.^58^ The ‘Practice Advisory’ status of this document means the recommendations are not supported by the same degree of scientific evidence as ASA guidelines or standards documents. Nevertheless, the Practice Advisory contains useful data and recommendations for consideration of local implementation. Most recommendations are based on consensus or low levels of evidence (Levels 4–5).

In certain circumstances (e.g. unusual patient positioning dictated by the demands of surgery or extremes of patient body habitus), it can be useful in the preoperative period to ask patients to self-position as they would for surgery. This process can usefully identify whether a particular position can comfortably be tolerated by an awake patient and may be most useful when ‘non-standard’ positions are proposed for prolonged surgery. If a proposed surgical position elicits discomfort in the upper limb, and particularly in the course of the ulnar nerve, then steps can be taken to mitigate this with a view to reducing the risk of PUN when surgery is subsequently undertaken.^58^

Key intraoperative measures to prevent PUN are summarised in Table 3. In all patients, this means positioning the arm to reduce pressure on the ulnar groove, preventing extremes of flexion at the elbow, and positioning the forearm in a supinated or neutral position when the patient is supine. Despite meticulous positioning of the upper limb during anaesthesia, avoidance of excessive elbow flexion or extension, and avoidance of external compression over the cubital tunnel, PUN can still result. Numerous case reports describe PUN despite adequate padding applied to the ulnar groove,^60^ and large registry database studies report that 18–27% of ulnar nerve injuries occur in cases where appropriate padding of the ulnar groove had been performed.^9^^,^^28^

**Table 3:** Summary of key intraoperative measures to prevent postoperative ulnar neuropathy (PUN).

Preventative advice	Rationale
Prevent excessive flexion of the elbow	Flexion greater than 90° may increase the risk of PUN and should be avoided unless the requirements of safe surgery necessitate it.
Ensure the forearm is in a supinated or neutral position when the arm is abducted during surgery	Forearm pronation increases pressure within the ulnar groove.
Ensure the forearm is in a neutral position when the arm is adducted (or ‘tucked’) at the side of the torso during surgery	Forearm pronation increases pressure within the ulnar groove.
Check that the distal edge of a blood pressure cuff is several centimetres above the medial epicondyle of the humerus	Placing the distal edge of an automatically cycling blood pressure cuff on the cubital tunnel induces ulnar paraesthesia in awake volunteers, and cases of postoperative ulnar neuropathy have been speculatively ascribed to this practice.^59^ If an upper limb has pre-existing peripheral neurological deficit, or is at higher risk of sustaining PUN than a contralateral limb during surgery because of requirements of positioning, it appears reasonable to place the blood pressure cuff on the unaffected limb.
Pad surgical surfaces in contact with the ulnar groove with foam or gel pads	Padding distributes the gravitational force applied, reducing pressure transmission to the contents of the ulnar groove. There is no evidence that any specific padding system is superior in terms of PUN risk reduction.

The relative contribution to ulnar nerve injury of events during the postoperative period (when the patient may no longer be under the direct care of the anaesthetist) is debated. The presentation of some cases of PUN days or weeks after surgery has a variety of possible explanations. Distracting events (such as surgical pain) in the immediate postoperative period may prevent a patient paying due regard to early symptoms. Patients may notice unusual ulnar symptoms but place little concern in them, given their overall priorities for functional recovery after surgery. Patients may raise concerns with staff regarding ulnar symptoms, but unless these staff are aware of the specific neuropathic phenomenon, these concerns may remain informal, undocumented, and may never be escalated to anaesthetic or neurological staff. It is also possible that a nervous injury presenting in the later postoperative period was itself sustained in that period and is causally unrelated to immediate perioperative care.^9^^,^^10^ It has been shown that the incidence of ulnar neuropathy is approximately the same in both surgical and medical in-patients,^61^ further supporting the hypothesis that PUN can arise from injurious events unrelated to anaesthetic or surgical care.

Documentation of the steps taken to reduce the risk of PUN in high-risk patients is important. In one analysis of closed claims of peripheral nerve injury after general anaesthesia, documentation was judged to be missing or erroneous and to be a contributory factor to the claim in 24% of cases.^62^ Patient positioning during anaesthesia and surgery is often documented in both medical (i.e. anaesthetic) and nursing (i.e. perioperative care practitioner) records. Despite this, or perhaps because of this, retrospective review of records typically yields incomplete or conflicting information about how patients were positioned and protected during anaesthesia. The anaesthetist carries overall responsibility for the physical safety of patients during anaesthesia, and therefore should satisfy themselves of the overall standards of record keeping kept during anaesthetic care. Minimum suggested intraoperative documentation regarding the prevention of PUN that we consider reasonable is listed in Table 4. In surgical procedures of prolonged duration (>2 h), local protocols should advise intermittent checks and repositioning of limbs. In the absence of such protocols, clinicians are advised to document the intermittent checks they perform during anaesthesia, acknowledging that surgical draping and the sterile field may reduce access to the patient to perform such inspection. In addition to these measures, prevention of systemic insults to nerve tissue (hypotension, hypoperfusion, etc.) is advisable.^15^

**Table 4:** Minimum suggested intraoperative documentation regarding the prevention of postoperative ulnar neuropathy (PUN).

Documentation	Comments
Intraoperative patient position	The overall position of the patient during surgery (i.e. supine, lithotomy, prone, etc.)
Position of the upper limbs	In relation to the torso (i.e. extent of shoulder adduction or abduction); vertical height of arms in relation to torso; extent of elbow flexion; position of forearms (i.e. supinated, neutral, or pronated)
Use of padding at the elbow	
Any specific intraoperative positioning actions taken to reduce risk of PUN	As indicated by increased risk of PUN identified at preoperative assessment
Any repositioning or intermittent checks of arm position performed during surgery	Likely to be of greatest relevance in surgery >2 h duration
Presence or absence of neurological abnormality in the recovery room	As indicated by increased risk of PUN identified at preoperative assessment

### Clinical presentation

Ulnar neuropathy at the cubital tunnel is characterised by paraesthesia or pain in the fourth and fifth digits or medial forearm. Weakness of flexor digitorum profundus, lumbricals, and intrinsic hand muscles may be present. The diagnostic value of Tinel's sign or flexion compression tests at the elbow is poor. Tinel's sign has a sensitivity, specificity, positive predictive value, and negative predictive value of 62%, 53%, 77%, and 30%, respectively; it therefore offers little benefit over routine sensory and motor neurological examination.^63^ Atrophy of the ulnar intrinsic hand muscles has been described after surgery.^64^ In lesions arising from the cubital tunnel, the flexor carpi ulnaris muscle may be unaffected.^42^ The differential diagnosis for paraesthesia in the fourth or fifth digit or hand grip weakness in the postoperative period should always include carpal tunnel syndrome, as this is a known mimic of ulnar neuropathy,^10^ also presenting with digital numbness, paraesthesia, and weakness. Carpal tunnel syndrome can usually be differentiated clinically because the compression of the median nerve affects the first to third digits.

### Natural history

Symptomatic ulnar neuropathy may not manifest in the recovery room or indeed for the first postoperative days.^65^ In the largest prospective examination of the condition published, none of the neuropathies identified were evident in the first two postoperative days.^10^ The same study reported that over half of ulnar neuropathies had resolved at 6 weeks. Nevertheless, presentation with pain or paraesthesia in an ulnar distribution has been described immediately after awaking from general anaesthesia.^25^ Accurately determining the time of onset of symptoms can be challenging, given the co-existence of acute post-surgical pain, strong narcotic analgesia, and sleep deprivation common in the first postoperative days after major surgery.^42^ The reported proportion of patients with PUN who go on to suffer permanent disability or non-disabling harm varies in the literature, but permanent features have been reported in as many as half of cases.^11^

### Diagnosis

Whereas the signs and symptoms of ulnar nerve dysfunction are straightforward to detect at the bedside, identifying the site, severity, and aetiology of the condition is more challenging. Neurological examination of the limbs and cranial nerves should be documented at the earliest opportunity in a patient suspected of having PUN. Routine screening of patients for evidence of PUN in the recovery room should not be undertaken.^66^ Sensory findings in PUN may include paraesthesia, numbness, pain, and loss of two-point discrimination in the ring and little finger.^26^

Broad differential diagnoses to consider in such cases are muscle diseases, radiculopathy, myelopathy, spinal cord trauma/infarction, and stroke.^67^ A brachial plexus lesion arising from compression on a cervical rib is a further differential diagnosis to consider. A pre-existing abnormality along the course of a peripheral nerve, for example an area of nerve compression, may increase the likelihood that a further physically distinct insult will manifest with signs of symptoms of nerve injury. This is known as the ‘double-crush’ phenomenon.^67^, ^68^, ^69^ A history of pre-existing neurological symptoms in the upper limb should, therefore, be sought. New, acute postoperative pain or paralysis should trigger assessment for compartment syndrome or acute vascular compromise.^70^ Communication to the patient of examination findings, possible diagnoses, and further assessment should be undertaken by the relevant anaesthetist and must be documented in the medical record.^87^ When notified that a patient has developed PUN (which may come to the attention of anaesthetists months or even years after anaesthesia), practitioners should express their sympathy and offer any assistance to patients. In UK practice, offering an apology when something goes wrong does not mean admitting legal liability and does not automatically mean taking personal responsibility for preceding events. Such communication should be copied to the patient's general practitioner/family doctor. It is imperative that the anaesthetist or surgical team or both ensure a responsible physician is involved in the patient's care until either PUN symptoms resolve or treatment or oversight of long-term dysfunction is established.

In conjunction with a neurophysiologist, a neurologist or peripheral nerve surgeon can advise on further clinical, electrophysiological, radiological, and tissue investigations. Patients with suspected PUN should be investigated for previously undiagnosed diabetes and other common causes of peripheral neuropathy, such as B12 deficiency and thyroid dysfunction.^67^ Electrodiagnostic studies (nerve conduction studies and needle motor and sensory EMG) are used to determine the site of the nerve lesion and estimate its severity,^42^ but their timing, execution, and interpretation are beyond the scope of this review. Bilateral nerve conduction testing should be undertaken, as the contralateral (unaffected) nerve will, in 85% of cases, display slowing of conduction, suggesting a predisposition to the neuropathy.^16^ The contralateral nerve cannot be considered a true ‘control’ because it will have been exposed to at least some of the same conditions of anaesthesia and surgery as the affected nerve^71^; however, it is known that many patients with PUN have a pre-existing subclinical neuropathy that is brought to clinical attention by perioperative events. The timing of electrodiagnostic studies is a complex area with no definitive guidance available to anaesthetists. EMG performed immediately postoperatively may assist in determining whether any neurological deficit was present before anaesthesia and surgery, as signs of denervation take more than 2 weeks to appear^18^; however, tests undertaken within 7–10 days of presentation can be falsely reassuring, as the processes of nerve degeneration will not yet manifest.^70^ Electrodiagnostic testing is often deferred until 3–4 weeks after injury because, by that time, a temporary neuropraxia will have resolved, and the studies will usually provide diagnostic and prognostic information.^15^^,^^72^ MRI of the brachial plexus can provide useful information on anatomical abnormalities at the level of roots, trunks, and cords to account for a patient's symptoms. Imaging can also usefully exclude the presence of neuroma in cases manifesting later in the postoperative period.^73^ Nerve biopsy can be undertaken on the advice of specialist neurological input to exclude an inflammatory component to the injury pattern.^74^ Serial electrodiagnostic testing, conducted every 3 months, is most useful to document the recovery of nerve function.^72^

### Management

**Fig 2:**
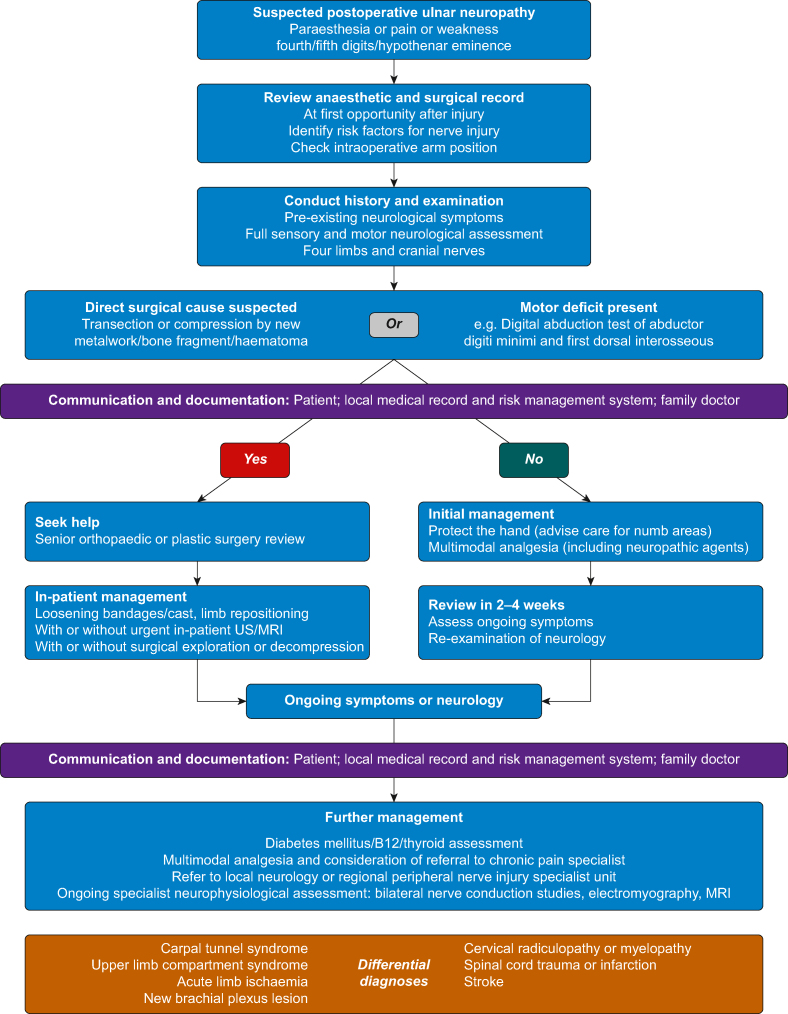
Proposed system of management for suspected postoperative ulnar neuropathy.

There is little published guidance to inform the management of PUN. The principles of management must therefore be extrapolated from the more extensive literature on the related fields of peripheral nerve injury caused by direct surgical trauma and suspected nerve injury after peripheral nerve block.^75^^,^^76^ Figure 2 outlines a proposed system of management for suspected PUN. At presentation, it is usually impossible to determine which lesions will subsequently reveal themselves as neuropraxic and those of axonotmesis or neurotmesis. A practical approach at presentation is, therefore, to determine whether or not in-patient investigative or surgical management is required. If direct surgical trauma is a likely cause to the injury, or if motor deficit is present, help should be summoned to determine if in-patient remedial action (including surgical re-exploration) is required. MRI and ultrasound scanning can be performed if compressive haematoma is a suspected cause for the patient's acute presentation. In the absence of a direct surgical cause or a motor deficit, an outpatient approach to management may be advocated. In cases of non-resolving symptoms, onward referral to a neurologist; plastic or peripheral nerve surgeon; and, if required, pain specialist is important after PUN. The anaesthetist is well placed to coordinate these referrals and to ensure that holistic support is provided to the affected patient. Prompt diagnosis and clear communication of a management plan are important to maintain patient–clinician trust.^70^^,^^77^ Delayed specialist consultation and delayed/missed diagnosis were contributory factors in 17% of peripheral nerve injury closed claims reported in one dataset between 1996 and 2015.^62^ Patients with PUN may experience pain with a negative impact on their quality of life.^78^ Impact on quality of life may be correlated with the degree of pain experienced and a co-existing diagnosis of depression. Severe or worsening neuropathic pain in suspected PUN should trigger clinical review, as it may indicate deteriorating nerve function because of untreated underlying mechanisms, such as ongoing nerve compression.^70^ Alongside pharmacological treatments for pain, a pain specialist will be well placed to engage with the psychosocial aspects of their pain experience. This may include formalised cognitive therapies but also informal encouragement to return to previous occupational and social activities.

Considerations for surgical intervention in cases of PUN are complex and expertly reviewed elsewhere.^72^ A distinction should again be drawn between cases of acute iatrogenic ulnar nerve injury attributable to surgical trauma to the upper limb (usually resulting in partial or complete nerve transection) and cases of PUN that manifest in the absence of obvious direct surgical insult. In the latter, whether a case of PUN will ultimately benefit from peripheral nerve surgery is determined by the nature and severity of the nerve injury, whether the overall post-injury clinical trajectory is one of improvement, the time elapsed since the injury was sustained, and patient factors (such as comorbidities). Regardless of the likely need for surgical intervention (indeed, the majority of patients sustaining PUN do not appear to undergo therapeutic surgery),^18^ the experience of peripheral nerve surgeons in assessing and managing nerve injury means that their input in cases of proven PUN is extremely helpful.

Rehabilitation therapies, including physiotherapy, after PUN will aim to educate patients, minimise functional decline, and improve sleep and psychological coping. These are best administered by specialist teams associated with nerve injury units, rather than non-specialist providers.

## Discussion

This review has summarised the current literature on PUN. A broad range of articles was returned by the search strategy, including case reports and case series, observational clinical studies, retrospective and registry analyses, and review or opinion pieces. PUN is rare, and the incidence is probably decreasing over time with general improvements in perioperative care. It is most often reported in men aged 50–75 yr, many of whom have an underlying subclinical ulnar neuropathy. A range of other factors may be associated with PUN, including a pre-existing peripheral neuropathy, history of cancer, extremes of body habitus, smoking, alcohol misuse, prolonged surgical duration, diabetes mellitus, and peripheral vascular disease. The anatomy of the ulnar nerve means that it is more frequently injured than the median or radial nerve. In approximately half of cases, signs and symptoms are temporary, resolving within weeks to months of presentation. Although guidance exists to reduce the risk of PUN, recommendations are based on low-quality evidence. Nevertheless, anaesthetists should document the steps taken to reduce risk during anaesthesia in all patients, including arm positioning and padding. In selected high-risk patients, further documentation of repositioning, intermittent checks, and neurological examination in the recovery room may be helpful. The management of PUN relies on adaption of related guidance on the management of peripheral nerve injuries in the perioperative period; a PUN management flowchart has been proposed (Fig. 2).

### Implications for practice and research

Although an individual anaesthetist may only encounter one or two cases of suspected PUN during their career, they should be familiar with the basic steps of diagnosis and management and, importantly, be responsible for coordinating timely onward referral. Multiple specialties, including acute pain services, orthopaedic surgery, plastic surgery, neurology, neurophysiology, chronic pain, and physiotherapy, may be consulted. Verbal and written communication to the patient is vital, and the responsible general practitioner/family doctor should be informed of the patient's condition.

Identifying individuals at highest risk of sustaining PUN is an area of current research. Intraoperative somatosensory evoked potential monitoring has been described in the context of prevention (or early detection) or perioperative nerve injury and PUN,^79^^,^^80^ but it remains unknown whether SSEP monitoring can decrease the incidence of PUN.^15^ The logistical implications of SSEP monitoring are a further barrier to its widespread use, although technical solutions are in development to make SSEP monitoring more feasible in a routine clinical environment.^81^^,^^82^ However, whether the procedure is of any utility in detection or prevention of PUN is unknown. In cases of proven PUN, the possible role of medical therapy with immunomodulating agents, such as methylprednisolone or i.v. immunoglobulin, in PUN is unclear, although both drugs have been used in cases of biopsy-proven post-surgical inflammatory nerve injury.^74^^,^^83^ Given the limited evidence base and possible harms associated with these agents, it would seem prudent that treatment should only be instituted after consultation with specialist neurology or regional peripheral nerve units.

## Conclusions

From the perspective of clinical anaesthetists, the literature on postoperative ulnar neuropathy supports the routine preoperative identification of known risk factors. Guidance on prevention of postoperative ulnar neuropathy is based on consensus and expert opinion, but it can be usefully incorporated into the day-to-day practice of clinicians. Particular attention should be paid to the conduct and documentation of protective measures because these are often delegated to non-anaesthetic operating theatre staff. A flowchart of postoperative ulnar neuropathy management has been proposed based on existing documents, with emphasis on patient and inter-specialty communication.

## Authors’ contributions

Review design/planning: DWH, JGH.

Review conduct, writing, and revision of paper: all authors.

## Declarations of interest

DWH and JGH accept fees for advising in civil, criminal, and coronial medicolegal cases. DWH is a member of the associate editorial board of the *British Journal of Anaesthesia*. JGH is the associate editor-in-chief of the *British Journal of Anaesthesia*. TK has no interests to declare.
