# Supplementary material for: Postoperative ulnar neuropathy: a systematic review of evidence with narrative synthesis
Source: Br J Anaesth. 2023 May 15;131(1):135–49. doi: 10.1016/j.bja.2023.04.010 (PMC10308442; doi:10.1016/j.bja.2023.04.010)
Supplement: Multimedia component 1 [file mmc1.docx]

***OVID Search Strategy (MEDLINE)***

***Downloaded***

**Ulnar nerve injury MEDLINE** Permanent

1

ulnar.mp. [mp=title, book title, abstract, original title, name of substance word, subject heading word, floating sub-heading word, keyword heading word, organism supplementary concept word, protocol supplementary concept word, rare disease supplementary concept word, unique identifier, synonyms]

00:01

2

limit 1 to humans

00:02

3

Ulnar Nerve/

00:01

4

limit 3 to humans

00:01

5

nerv*.mp. [mp=title, book title, abstract, original title, name of substance word, subject heading word, floating sub-heading word, keyword heading word, organism supplementary concept word, protocol supplementary concept word, rare disease supplementary concept word, unique identifier, synonyms]

00:01

6

limit 5 to humans

00:07

7

neuro*.mp. [mp=title, book title, abstract, original title, name of substance word, subject heading word, floating sub-heading word, keyword heading word, organism supplementary concept word, protocol supplementary concept word, rare disease supplementary concept word, unique identifier, synonyms]

00:01

8

limit 7 to humans

00:36

9

palsy.mp. [mp=title, book title, abstract, original title, name of substance word, subject heading word, floating sub-heading word, keyword heading word, organism supplementary concept word, protocol supplementary concept word, rare disease supplementary concept word, unique identifier, synonyms]

00:01

10

limit 9 to humans

00:01

11

injur*.mp. [mp=title, book title, abstract, original title, name of substance word, subject heading word, floating sub-heading word, keyword heading word, organism supplementary concept word, protocol supplementary concept word, rare disease supplementary concept word, unique identifier, synonyms]

00:01

12

limit 11 to humans

00:09

13

intra-operative.mp. [mp=title, book title, abstract, original title, name of substance word, subject heading word, floating sub-heading word, keyword heading word, organism supplementary concept word, protocol supplementary concept word, rare disease supplementary concept word, unique identifier, synonyms]

00:01

14

limit 13 to humans

00:03

15

intraoperative.mp. [mp=title, book title, abstract, original title, name of substance word, subject heading word, floating sub-heading word, keyword heading word, organism supplementary concept word, protocol supplementary concept word, rare disease supplementary concept word, unique identifier, synonyms]

00:01

16

peri-operative.mp. [mp=title, book title, abstract, original title, name of substance word, subject heading word, floating sub-heading word, keyword heading word, organism supplementary concept word, protocol supplementary concept word, rare disease supplementary concept word, unique identifier, synonyms]

17

limit 16 to humans

18

perioperative.mp. [mp=title, book title, abstract, original title, name of substance word, subject heading word, floating sub-heading word, keyword heading word, organism supplementary concept word, protocol supplementary concept word, rare disease supplementary concept word, unique identifier, synonyms]

19

limit 18 to humans

20

postoperative.mp. [mp=title, book title, abstract, original title, name of substance word, subject heading word, floating sub-heading word, keyword heading word, organism supplementary concept word, protocol supplementary concept word, rare disease supplementary concept word, unique identifier, synonyms]

21

limit 20 to humans

22

post-operative.mp. [mp=title, book title, abstract, original title, name of substance word, subject heading word, floating sub-heading word, keyword heading word, organism supplementary concept word, protocol supplementary concept word, rare disease supplementary concept word, unique identifier, synonyms]

23

limit 22 to humans

24

14 or 15 or 17 or 19 or 21 or 23

25

2 or 4

26

6 or 8 or 10 or 12

27

anaesth*.mp. [mp=title, book title, abstract, original title, name of substance word, subject heading word, floating sub-heading word, keyword heading word, organism supplementary concept word, protocol supplementary concept word, rare disease supplementary concept word, unique identifier, synonyms]

28

limit 27 to humans

29

anesth*.mp. [mp=title, book title, abstract, original title, name of substance word, subject heading word, floating sub-heading word, keyword heading word, organism supplementary concept word, protocol supplementary concept word, rare disease supplementary concept word, unique identifier, synonyms]

30

limit 29 to humans

31

24 or 28 or 30

32

25 and 26 and 31
